# Supplementary material for: How a co-design process led to more contextually relevant family planning interventions in emerging urban settings in Eastern Uganda
Source: PLOS Glob Public Health. 2023 Sep 29;3(9):e0002421. doi: 10.1371/journal.pgph.0002421 (PMC10540946; doi:10.1371/journal.pgph.0002421)
Supplement: S1 Table — These prototypes were aimed at addressing challenges on the demand-side, supply-side, and governance aspects. Out of the fourteen prototypes, eight were designed to target demand-side challenges, four to tackle supply-side challenges, and the remaining two prototypes focused on governance challenges. (DOCX) [file pgph.0002421.s001.docx]

**S1 Table: Prototypes developed by the participants for each of the design challenges**

| **Design challenge** | **Prototypes** |
| --- | --- |
| **Design challenge 1:**  How might we increase knowledge and understanding of voluntary Family Planning among women, men and young people in towns and cities? | **Prototype 1: ‘Izima’ or ‘Amazima ku famire planning’ [the truth about family planning] campaign**  Focus on dispelling myths about family planning (FP) and how to manage side effects. Leverage existing community structures to share information about FP, such as local leaders and trained Community Health Workers (CHWs). Also use mass media, social media platforms, information, education and communication (IEC) material and drama to disseminate the truth about FP. Target herbalists as well and provide them with information on facts about FP. This could be done through meetings organized for these. Lastly, community champions could also help disseminate information about FP. |
|  | **Prototype 2: Target parents to reach the youths and adolescents**  Parents should be encouraged to talk to their children about FP and sexuality in general. Leverage parents' meetings in schools. Also, use CHWs to share information on mobile and static megaphones in the markets where parents. Also reach them through social groups they subscribe too. |
|  | **Prototype 3: Target men and provide facts and information about FP**  Target men such as the *Boda-Boda* [motorcycle] riders at their stations through their leaders. Provide incentives to males who come with partners for FP, such as, T-shirts, helmets and reflector jackets. They could also be found at the workplaces especially in the informal business settings, for instance, garages, markets and bars. |
|  | **Prototype 4:** **‘Beeda or situla Kyosobola’ [carry what you can manage] campaign**  This campaign could be led by religious leaders in churches and mosques. Focus should primarily be on child spacing and having manageable families and not necessarily having few children. Also use the campaign to sensitize men about their responsibilities and what it means to have a large family size. |
|  | **Prototype 5: Community groups to promote uptake of FP in towns/urban centers?**  Rollout out ‘izima’ messages in the community groups using different channels. Use already existing women, youth groups, religious, savings and persons with disabilities (PWDs) groups among others. Use the leaders of those groups as facilitators. Plug into group meeting schedules instead of creating independent meeting days. Also engage CHWs to mobilize all members of these groups. |
|  | **Prototype 6: Use social media and digital technologies to promote behavior change and uptake of VFP**  Primarily use WhatsApp: Form WhatsApp groups for different categories of people based on age, gender, PWDs, social status, work environment and residence, among others. Groups should have multiple administrators to allow adding more members. Each group should have a health provider to answer the more technical questions. |
|  | **Prototype 7: Use Interpersonal (peer-to-peer) communication to promote FP uptake**  Train CHWs to provide FP methods that they are authorized to, such as, pills, condoms, and injectable contraceptives. Provide them with IEC materials developed for the ‘*Izima’* campaign. CHWs could be facilitated to use static and mobile megaphones to sensitize communities and to provide their contact details for inquiry in case of questions/clarifications. These should be availed transport means and provided with necessary tools, such as bags and reporting tools. The CHWs should be attached to public and private health facilities for referral in case of any side effects, easy supervision of their activities and for provision of commodities and supplies. |
|  | **Prototype 8: Provider-led counselling to promote uptake of voluntary FP**  Start by calling meetings with health facility owners (clinics, pharmacies, drug shops) and heads of the public facilities for buy-in. Owners or heads should then organize meetings with all health providers in the respective health facilities to introduce the idea. Conduct training to enhance provider-led counselling and integration of FP services into other health services. Continuous Medical Education (CME) should also be done to ensure skill retention. |
| **Design challenge 2:** How might we ensure consistent provision of quality voluntary family planning (VFP) services in emergent towns and cities? | **Prototype 1: Enhance knowledge and skills of providers to provide quality FP services through trainings and mentorships**  Identify at least 3 staff in each health facility to be trained on FP counselling and service provision through standalone workshops on a quarterly basis in the two sites. Trainings should be accompanied by regular support supervision. Form provider committees for quality improvement at city or municipal level. The committee should be mandated with quarterly support supervision, CMEs and provider mentorships. Trained staff should cascade skills to colleagues through CMEs. In big health facilities, also train providers that do not provide FP, for instance, those in Outpatients’ Department and immunization sections to promote integration of services. |
|  | **Prototype 2: Ensure affordable FP commodities are available in private clinics and at community level**  Conduct periodic outreaches in communities to provide free or subsidized FP services. Explore the possibility of obtaining free FP commodities from government supply chain to private facilities, drug shops and pharmacies to offer FP at affordable prices. |
|  | **Prototype 3: Create a platform for providers to learn and exchange ideas and information about FP**  Have a WhatsApp group of FP focal persons per facility to facilitate timely exchange of ideas and information on stock levels and availability of products in different health facilities. Use the SMS platform to target providers without smart phones. This platform should also include the urban health authorities. The group can also have CHWs, implementing partners and FP focal persons to provide FP related information and updates. Hold periodic physical dialogues or meetings with FP focal persons of facilities to understand the challenges, barriers and device solutions to challenges. |
|  | **Prototype 4: Timely management and referral for side effects management**  Each clinic to build a data base of clients who have received the different FP methods. A data base of clients served during outreaches or in the community should be built and attached to nearby private or public health facility for monthly follow-up for side effects management. Clinics or providers to be facilitated to make monthly voice calls to follow-up on clients on whether they are experiencing any side effects. Clients found experiencing side effects to be managed at no charge by the facilities that provided the methods. |
| **Design challenge 3:** How might leaders build capacity to lead or manage FP programs/create an enabling environment to improve organizational and management systems for FP provision? | **Prototype 1: Increase knowledge and awareness about FP among leaders: *‘Fuuka eyebuzibwaaku kubyafamire planning’* [Become someone who can be consulted about family planning] campaign**  Local council chairpersons, councillors, parish chiefs, sub-county chiefs, Community Development Officers, health assistants, mayors should be educated about FP. During the training, also use demonstrations as some leaders have never seen some of the methods. Focus on the mode of action of these methods to enable the leaders explain to their communities. Use expert trainers from the Ministry of Health or health workers focused on Reproductive Health. Provide certificates to trained leaders. Commission these leaders to use their platform to share information, for instance during burial ceremonies, parties and community meetings. |
|  | **Prototype 2: Orient technical and political leaders in planning, coordination and monitoring of FP activities in their areas**  Local council, sub county leaders, district leaders and district health teams should be trained on evidence-based planning, budgeting and accountability during FP provision. Empower leaders to demand/advocate for appropriate budgetary allocation to FP, accountability and right reports at all levels starting from the village to the district level |
