# Supplementary material for: How a co-design process led to more contextually relevant family planning interventions in emerging urban settings in Eastern Uganda
Source: PLOS Glob Public Health. 2023 Sep 29;3(9):e0002421. doi: 10.1371/journal.pgph.0002421 (PMC10540946; doi:10.1371/journal.pgph.0002421)
Supplement: Attachment — Submitted filename: Co-design manuscript_Response to reviewers_Final.docx [file pgph.0002421.s002.docx]

Makerere University School of Public Health

Kampala, Uganda

20^th^ July 2023

The Editor,

PLOS Global Public Health

**RE: Response to reviewer comments, Manuscript: PGPH-D-23-00486**

Thank you for considering our paper and providing feedback. We appreciate the opportunity to address reviewer queries and concerns and to improve our manuscript.

**Journal requirements**

**You indicated that you had ethical approval for your study. In your Methods section, please ensure you have also stated whether you obtained consent from parents or guardians of the minors included in the study or whether the research ethics committee or IRB specifically waived the need for their consent.**

Thank you for bringing this to our attention. It is important to note that for all minors (excluding emancipated minors), written informed consent to participate in the formative research and co-design workshop was obtained from their household heads. This has also been included under the ‘Ethical considerations’ section on Page 15, Lines 290-291).

**We have noticed that you have uploaded Supporting Information files, but you have not included a list of legends. Please add a full list of legends for your Supporting Information files after the references list.**

Thank you for your guidance. The list of legends has been added on Page 43, as requested.

**Reviewer #1:**

**This is an exemplary co-design approach for other projects that are being implemented in developing countries to design and develop context-specific interventions on FP programs. As this HCD is an emerging approach to most developing countries’ projects, this article will have a significant positive contribution to improving the practices of project/program design techniques for different related initiatives. The project team is expected for implementing the identified interventions and will share the outcomes of the intervention with the global communities at the end of the project.**

We thank the reviewer for their motivating compliment.

As part of our project dissemination plan, we are committed to sharing the findings on the effectiveness of the implemented interventions through a peer-reviewed article.

**Reviewer #2:**

**The analysis presented focuses on solution finding methodology described in through manner. Study design is well prepared and research was carried out with lose attention to the practices on the ground. A few limitations of the study which possibly exist should be reflected in the design or methodology in order to have better picture for the replicability of the study results.**

We appreciate the reviewer’s kind compliments.

Study limitations, including the limited sample size and the representativeness of the sample have been added at the end of the discussion section.

**Reviewer #3:**

**Thank you for the submission.
The paper is interesting to read. This is a well-written manuscript on an important topic. The methodology is scientifically sound and rigorous. Layout of results is incredible.**

We thank the reviewer for the positive feedback and appreciate the kind words.

**Reviewer #4:**

**The authors describe in detail the different stages of the co-design process leading to contextually relevant family planning interventions. They claim this process will lead to more relevant interventions for specific urban sub-populations.
Because the paper does not describe a research or research methods it cannot be considered for publication in PLOS Global Public Health, the first publication criterion being: ''PLOS Global Public Health is designed to communicate original research and research methods. We welcome public health research that reaches across disciplines and regional boundaries to address the biggest health challenges and inequities facing our society today.''**

We want to assure the reviewer that our manuscript makes a significant contribution to understanding the design and implementation of interventions to address real context-specific problems, which is crucial in global health research. We would like to point out that global public health encompasses not just analytical approaches, but also the synthesis of implementation research. In this regard, we strongly believe that our paper meets the publication criteria of PLOS Global Public Health as it effectively communicates an original approach to implementation research.

Our study highlights the application of the co-design methodology in low-resource urban settings, utilizing human-centered design principles that have been underutilized in the field of public health in developing countries. By employing this innovative methodology, we demonstrate the effectiveness and relevance of co-design in creating interventions tailored to the specific needs of family planning clients in these settings. This aspect of our study makes a valuable contribution to the existing knowledge in the field.

Furthermore, the insights gained through the co-design process have significant implications for the development and implementation of future interventions in similar settings. We firmly believe that the innovative methodology and the resulting findings make a valuable and distinct contribution to the field of global public health, aligning with the journal's mission to publish research that is methodologically and ethically rigorous.

**The authors could submit the paper to another journal, because the detailed description of the co-design process and the lessons drawn are useful for organisations developing FP interventions.
The following comments and suggestions could improve the paper (when submitted to another journal). In the introduction the authors argue that it is important to address the unmet need for FP for different sub-populations in urban areas, in particular the poor and other vulnerable populations, because there are intra-urban inequalities. (line 93-101). However, in the description of the process, there is no referral to different sub-populations, except by gender and age-group. Following the introduction one would expect address of socio-economic differences.**

We are taken aback by the reviewer's disregard for family planning as a global public health issue. We strongly believe that addressing the unmet demand for family planning requires innovative approaches that can help the global health community understand and tackle the challenges. We want to emphasize that this manuscript focuses on implementation research, demonstrating how we are utilizing human-centred design to address the challenges of family planning.

We appreciate the reviewer’s feedback regarding the inclusion of socio-economic differences in our study. We acknowledge that it would have been valuable to capture data on the socio-economic characteristics of the participants to provide a more comprehensive understanding of the specific sub-populations, particularly those who are poor or vulnerable, and to address the intra-urban inequalities mentioned in the introduction. Unfortunately, we did not to collect specific socio-economic information from the participants. However, it is worth noting that during the participant selection process, we made deliberate efforts to ensure representation from diverse locations and backgrounds, including both slum and non-slum settings. Additionally, we specifically included participants from fishing communities, who are known to face significant marginalization.

**Stage one of the co-design process is a mixed-methods study to understand the problem. The authors describe (203-204) that "data collected from the survey and qualitative interviews were analysed by the research team and discussed into actionable problem statements of focus during ideation". A summary of these findings by sub-population could be presented and reference made to the study report (just the study protocol is in the reference list (#31). The data should be made available.**

We acknowledge the potential value of including these findings in the manuscript to enhance the understanding of the context and challenges we aimed to address. However, because, at the time of writing, the findings had not yet undergone the rigorous peer review process, we refrained from including them in the present manuscript. Nevertheless, we have added a table summarizing the preliminary findings presented to the participants (see Table 2), and the pre-print manuscript that underpins the quantitative findings has been duly cited.

**The authors could explain how the participants for the co-design workshop were selected and which sub-populations the 13 community members represented (Line 212). A table with background of the 52 (total count not 56) workshop participants would be helpful. The same applies to the selection of participants of the test phase (line 254).**

We thank you for bringing to our attention the oversight regarding the total number of participants. We apologize for any confusion caused. The correct total number of participants was indeed 56, as previously stated. However, it should be noted that an additional participant category was unintentionally omitted. Four participants were selected from partner organizations actively involved in family planning initiatives within the two sites. This has been included on Page 11, under the “Define” section. Additionally, a description of how community members were selected and the distribution by age group and gender is provided on Page 11, under the “Define” section. The process of selecting participants in the test phase has also been provided on Pages 14 and 15, under the “Test” section.

**It would be interesting to present how the different groups of participants reacted to the key-insights from the study – were these new/surprising to them? (Table 2, line 302)**

We appreciate this feedback and have included a description of the participant reactions to the formative study findings on Page 17. Overall, the participants were in agreement with the findings presented (Table 2), but we obtained insightful comments from the religious leaders regarding what methods are permitted. The participants also expressed surprise at the higher family planning usage among mainland slum residents compared to those from non-slum settings, speculating that this could be due to the presence of marginalized poor populations in these areas, such as domestic workers.

**Table 3 (Line 308): Add to Design challenge # in first column the descriptions: Demand challenges, Supply challenges, Governance challenges (308)**

We thank the reviewer for this input. Design challenge descriptions have been added in Table 3 (Now Table 4). **Most likely, within the community participant group there have been power imbalances, by gender, age, education, etc.). Could you elaborate what you observed in the workshop? You say in conclusion: (612-615) ''For the success of the process, it is important to pay attention to participant characteristics, including the differences in education levels and position in the community, and how these could influence group dynamics and the quality of the discussions and to put in place measures to minimise the effects of this on the process.''**

We did not observe significant disparities in participation between men and women within the community group. However, it was noteworthy that adolescents in this group were less vocal. This could be attributed to the fact that there were only two adolescents present. We have included this observation in the findings section on Page 17, under the section titled “Group dynamics”. We have also included a paragraph discussing this finding with recommendations on Page 35-36.

**The practical suggestions for interventions by the workshop participants in Supporting file 1 could be more explored and commented on considering feasibility and being innovative and unexpected.**

We thank the reviewer for this comment. In response, we have duly acknowledged the absence of evidence regarding the feasibility of the developed interventions in the discussion section on Page 35. Furthermore, we have included a recommendation for conducting further testing to ascertain the feasibility, effectiveness, and sustainability of these interventions.

**In discussion and limitations be critical of:
• Whether the small number of participants represent the communities and health workers and policy makers in the two project areas.
• In-group power differences that may have influenced the co-design process: by gender, age.**

We appreciate this input and acknowledge that the small sample size in each participant group may have impacted the representativeness of perspectives from the diverse urban sub-populations. Additionally, this limitation could have influenced the level of participation, particularly among the adolescents. We have duly acknowledged these limitations in our methodology, highlighting the potential impact on the interventions delivered and the extent to which they address the diverse needs of all urban populations. This has been included on Page 36.

**Present study findings (of phase one) on the gaps in knowledge and skills of health workers. The interventions are heavy on provider training. (Line 428) Explore reasons why they do not disclose all side effects (Line 523)– this may be intentional and not because of gaps in knowledge or skills.**

The formative study findings regarding provider-client interactions and other supply-side barriers have been incorporated into Table 2.

**In discussion: Highlight the proposed unexpected and new interventions that are probably especially effective for urban areas (compared to rural areas).**

We thank the reviewer for this insightful comment. We concur that certain interventions proposed are better suited to urban settings, such as leveraging social media groups to enhance health worker communication. Additionally, the suggestion to pay more attention to community health worker motivation to ensure effectiveness of their activities, especially in urban contexts where people depend on daily income for their livelihood. We have taken this feedback into consideration and added corresponding remarks in the discussion on Page 33.

**It would be interesting to see (in future) the research paper on the effects of the interventions that were co-designed. (see line 615-19) ''The intervention model prioritized for implementation will undergo further adaptations, in line with the human-centred design process, and rigorous testing to assess its effect on the uptake of voluntary FP and the reduction of unmet need for FP in emergent urban settings in Eastern Uganda.''**

At the end of the project, we aim to publish manuscripts of both the outcome evaluation, describing what worked, and the process evaluation, detailing how the context and implementation affected the effectiveness of the interventions to further share our learnings. This has been added to the conclusion on Page 37.
